# Acellular Dermal Matrix (WITHderm^®^) Spacer Grafts for the Prevention of Lower Eyelid Ectropion After Subciliary Approaches in Facial Fracture Surgery: A Preliminary Study

**DOI:** 10.3390/jfb17040196

**Published:** 2026-04-18

**Authors:** Wooseob Kim, Eun A. Jang, Kyu Nam Kim

**Affiliations:** Department of Plastic and Reconstructive Surgery, Kangbuk Samsung Hospital, School of Medicine, Sungkyunkwan University, Seoul 03181, Republic of Korea; wskim2404@naver.com (W.K.); insane.blood.a@gmail.com (E.A.J.)

**Keywords:** subciliary approach, lower eyelid ectropion, acellular dermal matrix, spacer graft, facial fracture surgery

## Abstract

**Background/Objectives:** The subciliary approach offers excellent exposure for orbital and zygomaticomaxillary complex fracture repair but is associated with a relatively high risk of postoperative lower eyelid ectropion. This study evaluated the preventive efficacy of an acellular dermal matrix (ADM; WITHderm^®^) spacer graft placed during subciliary incision repair. **Methods:** This prospective observational cohort study included 20 patients who underwent open reduction and internal fixation for orbital wall or zygomaticomaxillary complex fractures using a subciliary approach between June and December 2024. A human-derived ADM (WITHderm^®^) spacer graft was interposed between the orbital septum and the orbicularis oculi muscle during incision closure. Postoperative outcomes were assessed at three time points: ectropion grading at 1 month and scar outcomes at 3 and 6 months using the Patient and Observer Scar Assessment Scale (POSAS). **Results:** No patients developed postoperative lower eyelid ectropion at 1-month follow-up (0% incidence). Both patient-reported and observer-reported scar outcomes improved significantly over time. The mean total PSAS score decreased from 21.0 ± 2.85 at 3 months to 11.3 ± 2.13 at 6 months (*p* < 0.001), while the mean total OSAS score decreased from 21.35 ± 2.25 to 11.4 ± 1.67 (*p* < 0.001). Overall patient satisfaction and objective scar ratings also showed significant improvement. **Conclusions:** ADM (WITHderm^®^) spacer grafting during subciliary incision repair appears to be a safe and effective strategy for preventing early postoperative lower eyelid ectropion and achieving favorable scar outcomes. Further studies are warranted to confirm these findings.

## 1. Introduction

The subciliary incision is widely used for exposure during orbital floor and zygomaticomaxillary complex (ZMC) fracture repair [1,2,3,4]. It offers broad visualization of the infraorbital rim and orbital contents but is associated with postoperative lower eyelid malposition, including scleral show, retraction, and ectropion [5,6,7,8]. Reported ectropion incidence ranges from 6% to 20% in standard subciliary approaches [5,6,7,8]. Ectropion arises from vertical shortening of the anterior lamella, scarring of the pretarsal orbicularis oculi, and cicatricial contracture [9,10,11]. Preventive strategies include Frost sutures, canthopexy, and meticulous pretarsal dissection [10,11,12,13,14]; however, no method fully eliminates the risk.

Acellular dermal matrix (ADM) grafts have gained attention in eyelid reconstruction and cosmetic lower blepharoplasty as spacer grafts because they restore vertical eyelid length, provide structural support, reduce scar contracture, and promote neovascularization and soft tissue remodeling [15,16,17,18,19,20,21]. ADM is a biologic scaffold derived from decellularized human or animal dermis, in which cellular components are removed while preserving the native extracellular matrix architecture, including collagen, elastin, and vascular channels [17,20]. This structural preservation allows ADM to serve as a biocompatible framework for host cell infiltration, revascularization, and tissue integration [17,20]. The advantages of ADM include low immunogenicity, favorable handling characteristics, and the ability to support tissue regeneration and remodeling, making it particularly suitable for delicate periorbital applications [17]. In addition, ADM can function as a mechanical spacer and interpositional barrier, which may help prevent scar contracture and maintain soft tissue contour [17,18,19]. However, ADM also has several limitations. These include additional cost compared with autologous or no graft use, potential variability in resorption or integration depending on the product and implantation site, and a theoretical risk of infection or inflammatory response, although reported complication rates are generally low in clinical practice [19]. Evidence from oculoplastic literature demonstrates ADM efficacy in cicatricial ectropion repair, lower eyelid retraction, and tear trough augmentation [18,19,20,21,22]. For examples, previous studies suggested that ADM spacer grafts used effectively for lower eyelid retraction repair [18,19]; ADM restoring attenuated orbital septa in blepharoplasty, maintaining contour and preventing contraction [20,21]; Porcine ADM (Enduragen^®^) used in 129 eyelids with significant symptom improvement and low complication rates [15]; and ADM use in tear trough deformity showing structural soft tissue augmentation [21,22].

However, to the best of our knowledge, no prior study has evaluated ADM spacer grafts as a preventive tool against ectropion in facial fracture surgery using subciliary incision. From this perspective, the present study aims to fill this gap by prospectively evaluating surgical outcomes of ADM interposition between the orbital septum and orbicularis muscle during subciliary incision for fracture repair.

## 2. Materials and Methods

### 2.1. Ethical Compliance

The Institutional Review Board (IRB) of Kangbuk Samsung Hospital (approval number: 2024-04-022; date of approval: 25 March 2024) approved this study. All research procedures followed the ethical guidelines outlined in the 1975 Declaration of Helsinki [23]. Informed consent was obtained from all subjects involved in the study. Written informed consent has been obtained from the patients to publish this paper.

### 2.2. Study Design and Patient Selection

This prospective observational cohort study was conducted at a single center between June and December 2024, following Institutional Review Board approval obtained prior to data collection. The acellular dermal matrix used in this study was a human-derived acellular dermal matrix (WITHderm^®^, REGENBIO Co., Ltd., Gimpo, Republic of Korea). WITHderm^®^ is composed of 100% human acellular dermal tissue processed to remove immunogenic cellular components while preserving the native dermal extracellular matrix structure.

Inclusion criteria were as follows: 1. Patients who underwent open reduction and internal fixation for ZMC or orbital wall fractures using a subciliary approach. 2. Placement of an ADM (WITHderm^®^) spacer graft at the time of subciliary incision repair.

Exclusion criteria were as follows: 1. History of prior lower eyelid surgery. 2. Presence of pre-existing eyelid malposition. 3. Severe soft-tissue injury around the eyelid associated with facial fractures.

### 2.3. Surgical Techniques (Subciliary Approach and Subciliary Incision Repair with WITHderm^®^ Spacer Graft)

**Figure 1:**
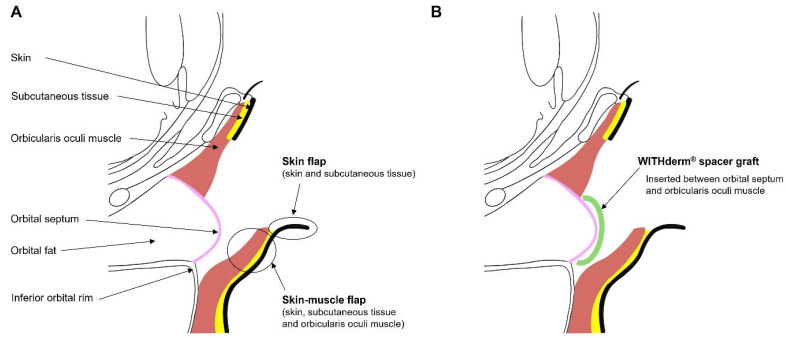
Schematic illustration of the subciliary approach and placement of the acellular dermal matrix (WITHderm^®^) spacer graft. (**A**) The subciliary approach. A skin flap consisting of the skin and subcutaneous tissue is initially elevated inferiorly, followed by elevation of a skin–muscle flap including the orbicularis oculi muscle. Dissection proceeds in the preseptal plane to the anterior edge of the infraorbital rim, allowing periosteal incision and subperiosteal exposure for fracture repair. (**B**) Subciliary incision repair with a WITHderm^®^ spacer graft. The acellular dermal matrix spacer graft is inserted between the orbital septum and the orbicularis oculi muscle, functioning as an interpositional spacer to prevent vertical anterior lamellar shortening and cicatricial contracture.

A subciliary incision was made approximately 2 mm below the lash line. A skin flap consisting of the skin and subcutaneous tissue was first elevated inferiorly for approximately 4–6 mm. This was followed by elevation of a skin–muscle flap, including the skin, subcutaneous tissue, and orbicularis oculi muscle, and dissection proceeded inferiorly in the preseptal plane to the anterior edge of the infraorbital rim (Figure 1A). At the infraorbital rim, the periosteum was incised and subperiosteal dissection was carried out to allow fracture exposure and open reduction and internal fixation.

**Figure 2:**
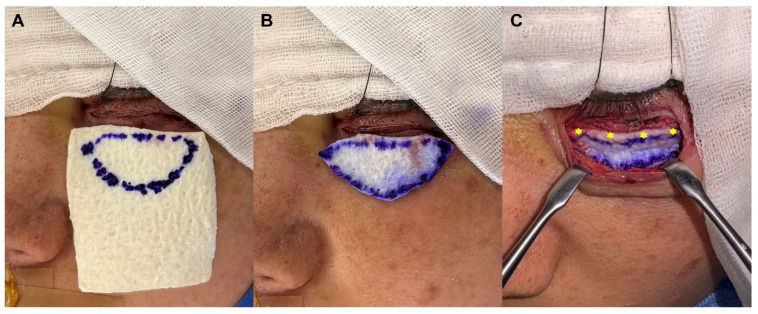
Clinical photographs demonstrating placement of an acellular dermal matrix (WITHderm^®^) spacer graft during subciliary incision repair. (**A**,**B**) WITHderm^®^ was trimmed into an elliptical shape to match the width of the lower eyelid and the exposed height of the orbital septum. (**C**) The WITHderm^®^ spacer graft was inserted between the orbital septum and the orbicularis oculi muscle and anchored to the orbicularis oculi muscle using 6-0 Vicryl interrupted sutures at four fixation points (yellow star marks).

During closure of the subciliary incision, an ADM (WITHderm^®^) graft was inserted between the orbital septum and the orbicularis oculi muscle as a spacer to prevent vertical anterior lamellar shortening and cicatricial contracture (Figure 1B). The graft was first trimmed into an elliptical shape to match the width of the lower eyelid and the exposed height of the orbital septum (Figure 2A,B). The superior margin of the trimmed graft was then anchored to the orbicularis oculi muscle using 6-0 Vicryl interrupted sutures at four fixation points (Figure 2C). Skin closure was subsequently performed with 6-0 nylon sutures.

### 2.4. Evaluation of the Postopertive Outcomes

We evaluated postoperative outcomes at three time points: ectropion grading at 1-month, short-term scar outcomes at 3 months, and long-term scar outcomes at 6 months.

The degree of ectropion at 1 month postoperatively was assessed using the lower eyelid ectropion grading scale shown in Table 1. This grading scale for lower eyelid ectropion was based on a severity-based clinical grading system modified from previous oculoplastic and facial trauma literature, incorporating the degree of scleral show, margin and punctal eversion, conjunctival exposure, and the need for surgical correction [14,24,25,26].

The Patient and Observer Scar Assessment Scale (POSAS) was used to evaluate short-term and long-term scar outcomes at 3 and 6 months postoperatively, respectively. For the Patient Scar Assessment Scale (PSAS), each patient rated six scar characteristics—pain, itching, color, stiffness, thickness, and irregularity—on a 10-point scale [27,28]. After completing these items, patients rated the overall scar appearance using a 10-point visual analog scale (VAS) to determine the overall patient satisfaction (OPS), with higher scores indicating poorer scar quality [27,28]. A single observer (the senior author) assessed the scars using the Observer Scar Assessment Scale (OSAS), which evaluates six parameters—vascularity, pigmentation, thickness, pliability, relief, and surface area—each scored on a 10-point scale [27,28]. The observer then rated the overall scar appearance using a 10-point VAS to obtain the objective scar rating (OSR), with higher scores representing worse scar appearance [27,28]. For both the PSAS and OSAS, total scores range from 6 to 60, corresponding to perfectly normal skin (score of 6) to the worst imaginable scar (score of 60) [27,28].

## 3. Results

A total of 20 patients (15 men and 5 women) who met inclusion and exclusion criteria between June and December 2024 were enrolled in the present study. Table 2 summarizes the clinical data of patients in this study. Mean patient age was 50.35 ± 16.24 years (ranged from 22 to 73 years), and facial fracture types included ZMC and orbital wall fractures in 16 and 4 cases, respectively.

### 3.1. Ectropion Outcomes

At 1-month follow-up, all patients showed ectropion degree of Grade 0, which means no scleral show and no eyelid margin eversion. Thus, there were no postoperative ectropion developments in this study (0% ectropion rate).

### 3.2. POSAS Outcomes

Table 3 shows POSAS data of patients in the present study. At 3-month follow-up, which means the short-term scar outcomes, the mean total PSAS, OSAS, OPS and OSR scores were 21.0 ± 2.85, 21.35 ± 2.25, 4.09 ± 1.02 and 4.19 ± 1.05, respectively. At 6-month follow-up, which means the long-term scar outcomes, the mean total PSAS, OSAS, OPS and OSR scores were 11.3 ± 2.13, 11.4 ± 1.67, 2.37 ± 0.70 and 2.46 ± 0.65, respectively.

Table 4 shows changes in patient-reported scar outcomes, including each item of PSAS and OPS between 3 and 6 months after surgery. Mean scores for all individual PSAS domains, including pain, itching, color, stiffness, thickness, and irregularity, were significantly lower at 6 months compared with 3 months (all *p* < 0.001). The mean total PSAS score decreased from 21.0 ± 2.85 at 3 months to 11.3 ± 2.13 at 6 months (*p* < 0.001), indicating progressive scar maturation and improvement. The mean overall patient satisfaction score decreased from 4.09 ± 1.02 at 3 months to 2.37 ± 0.70 at 6 months (*p* < 0.001), reflecting increased patient-perceived scar quality during long-term follow-up.

Table 5 shows changes in observer-assessed scar outcomes, including each item of OSAS and ORS between 3 and 6 months after surgery. Mean scores for all OSAS domains—including vascularity, pigmentation, thickness, pliability, relief, and surface area—were significantly lower at 6 months compared with 3 months (all *p* < 0.001). The mean total OSAS score decreased from 21.35 ± 2.25 at 3 months to 11.4 ± 1.67 at 6 months (*p* < 0.001), indicating substantial improvement in observer-assessed scar quality over time. Similarly, the OSR showed significant improvement, with mean scores decreasing from 4.19 ± 1.05 at 3 months to 2.46 ± 0.65 at 6 months (*p* < 0.001), reflecting favorable objective scar maturation during long-term follow-up.

**Figure 3:**
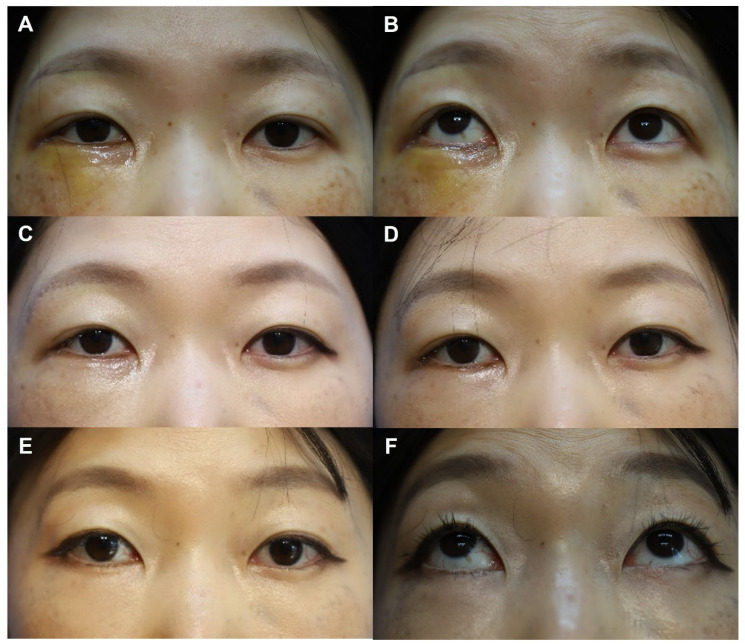
Clinical photographs of a 44-year-old female patient who underwent subciliary incision repair with a WITHderm^®^ spacer graft during open reduction and internal fixation of a zygomaticomaxillary complex fracture. (**A**,**B**) Ten days after facial fracture surgery. (**C**) One month after facial fracture surgery. No lower eyelid ectropion was observed (postoperative ectropion grade 0). (**D**) Three months after facial fracture surgery. The total Patient Scar Assessment Scale (PSAS) and Observer Scar Assessment Scale (OSAS) scores were 22 and 21, respectively. The Overall Patient Satisfaction (OPS) and Objective Scar Rating (OSR) scores were 5 and 4, respectively. (**E**,**F**) Six months after facial fracture surgery. The PSAS and OSAS scores were 12 and 13, respectively, while the OPS and OSR scores were both 3.

**Figure 4:**
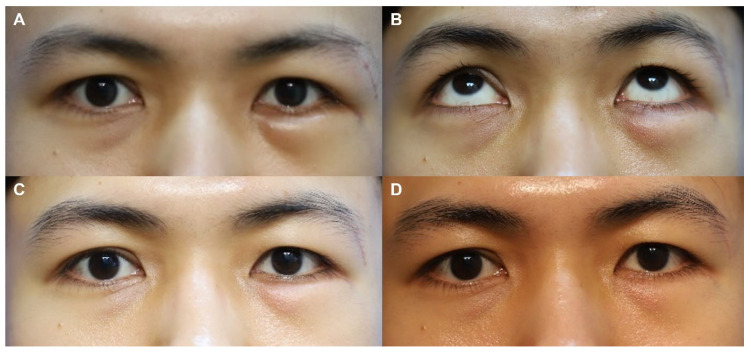
Clinical photographs of a 29-year-old male patient who underwent subciliary incision repair with a WITHderm^®^ spacer graft during open reduction and internal fixation of a zygomaticomaxillary complex fracture. (**A**,**B**) One month after facial fracture surgery. No lower eyelid ectropion was observed (postoperative ectropion grade 0). (**C**) Three months after facial fracture surgery. The total Patient Scar Assessment Scale (PSAS) and Observer Scar Assessment Scale (OSAS) scores were 17 and 23, respectively. The Overall Patient Satisfaction (OPS) and Objective Scar Rating (OSR) scores were 4 and 5, respectively. (**D**) Six months after facial fracture surgery. The PSAS and OSAS scores were both 8, while the OPS and OSR scores were both 2.

Figure 3 and Figure 4 present representative cases from this study, demonstrating serial postoperative follow-up clinical photographs.

## 4. Discussion

The present prospective observational study demonstrates that the use of WITHderm^®^ spacer graft during subciliary incision repair in facial fracture surgery was associated with zero cases of postoperative lower eyelid ectropion. This finding is clinically meaningful given the consistently reported rates of ectropion following traditional subciliary approaches in the existing literature [5,6,7,8,11]. In addition, patient- and observer-reported scar outcomes showed significant improvement between short-term and long-term follow-up, suggesting favorable soft-tissue healing and scar maturation when ADM is incorporated into subciliary incision repair.

### 4.1. Comparison with Existing Literature: Ectropion Prevention

Previous studies have consistently identified the subciliary approach as carrying the highest risk of postoperative lower eyelid malposition among commonly used lower eyelid incisions [5,6,7,8]. Ridgway et al., in a meta-analysis comparing lower eyelid approaches for orbital fracture repair, reported an ectropion rate of approximately 14% following subciliary incisions, compared with 1.5% for transconjunctival approaches [5]. Similarly, Neovius et al. reported an ectropion incidence of 8.1% in patients undergoing subciliary approaches despite the routine use of Frost sutures, indicating that conventional preventive measures may be insufficient [6]. Trevisiol et al. also documented an ectropion rate of 8.11% in their subciliary cohort [8].

In contrast, no cases of ectropion were observed in the present study, despite the known susceptibility of the subciliary approach to anterior lamellar shortening and cicatricial contracture [10,11,26]. This stark difference suggests a robust preventive effect of ADM spacer grafting when placed between the orbital septum and the orbicularis oculi muscle at the time of incision repair. While differences in study design, sample size, and follow-up duration must be considered, the absence of ectropion in our cohort compares favorably with historical controls reported across multiple independent studies [5,6,7,8].

### 4.2. Biological Rationale for ADM Spacer Grafting

The observed preventive effect of ADM spacer grafting is biologically plausible and supported by prior reconstructive and aesthetic eyelid literature [15,16,17,18,19,20,21]. ADM serves several complementary functions that are particularly relevant to subciliary incision repair. First, it acts as a vertical spacer, restoring and maintaining lower eyelid height by counteracting postoperative vertical shortening of the anterior lamella [17,20]. Second, ADM functions as a scar-modulating interpositional barrier, reducing direct adhesion between the orbital septum and orbicularis oculi muscle and thereby minimizing cicatricial tethering [17,20,29]. Third, ADM provides a biologic scaffold for vascular ingrowth and tissue remodeling, which may soften scar contracture and improve long-term tissue compliance [20,29].

These mechanisms are consistent with findings reported in oculoplastic surgery. A previous study has documented the efficacy of ADM spacer grafts in the treatment of cicatricial lower eyelid retraction, demonstrating improvements in eyelid position and symptom relief [20]. In aesthetic lower blepharoplasty, ADM has been shown to preserve the orbital fat position and reduce postoperative scarring by restoring attenuated orbital septa [21,22]. Furthermore, a previous study reported favorable outcomes and low complication rates in a large cohort of 129 eyelids treated with porcine ADM spacer grafts, supporting the safety and durability of ADM in periorbital applications [15]. The present study extends these therapeutic concepts to a preventive application in facial trauma surgery, where ADM is used proactively to mitigate known risk factors for ectropion.

While the concept of spacer grafting for lower eyelid support is well established in reconstructive and aesthetic contexts, its application as a preventive adjunct during primary subciliary incision repair in facial fracture surgery has been rarely reported. In this regard, the present study does not introduce a fundamentally new biological principle, but rather translates and adapts an established reconstructive concept into a preventive strategy within a trauma-specific surgical setting. This shift from therapeutic to prophylactic use, particularly in the context of acute facial fracture management, represents a clinically meaningful extension of existing knowledge and may provide a practical framework for reducing ectropion risk in high-risk surgical approaches.

### 4.3. Scar Outcomes and Soft-Tissue Healing

In addition to ectropion prevention, both patient-reported and observer-reported scar outcomes improved significantly between 3 and 6 months postoperatively. The reduction in PSAS and OSAS scores suggests progressive scar maturation and improved scar quality over time. These findings support the hypothesis that ADM interposition may reduce subciliary scar stiffness and limit downward traction forces acting on the lower eyelid margin [30].

Traditional subciliary incisions have been associated with visible scarring, pigmentation changes, and contour irregularities, as described by several previous studies [30,31,32]. In comparison, the favorable POSAS trends observed in this study suggest that ADM spacer grafting may contribute not only to functional protection against ectropion but also to improved aesthetic outcomes. This dual benefit is particularly relevant in facial fracture patients, for whom both functional recovery and cosmetic appearance are important determinants of overall satisfaction.

### 4.4. Clinical Significance

Lower eyelid ectropion, even when mild, can significantly affect patient quality of life through symptoms such as epiphora, ocular irritation, cosmetic asymmetry, and, in severe cases, corneal exposure and keratopathy [11,14,26]. These sequelae are especially impactful in facial fracture patients, who are often relatively young and cosmetically sensitive [11]. Importantly, the treatment of established ectropion frequently requires secondary surgical interventions, including lateral tarsal strip procedures, canthoplasty, or full-thickness skin grafting [10,11,12,13,14].

Cho et al. reported that severe ectropion following subciliary open reduction and internal fixation developed at a mean of 0.78 months postoperatively and required surgical correction at approximately 0.91 months, most commonly using lateral tarsoplasty combined with full-thickness skin grafting [11]. Such secondary procedures increase patient morbidity, prolong recovery, and add healthcare costs. From this perspective, prevention of ectropion may be more valuable than delayed correction, and the routine use of an ADM spacer graft during subciliary incision repair may represent a proactive strategy to reduce the need for secondary reconstructive surgery.

### 4.5. Limitations of the Present Study

Several limitations of this study should be acknowledged. Importantly, this study was designed as a preliminary observational investigation to explore the potential role of ADM spacer grafting in ectropion prevention, and the findings should be interpreted within this exploratory context. First, the sample size was relatively small (n = 20), which limits the statistical power and generalizability of the findings. Given the limited number of patients, the absence of observed ectropion in this cohort should be interpreted with caution, as the study may be underpowered to detect low-frequency complications. In addition, no a priori power calculation was performed, as this study was designed as a preliminary exploratory investigation. The relatively small sample size further limits the statistical robustness of the findings. Notably, although no cases of ectropion were observed (0/20), the 95% confidence interval for the incidence ranges from 0% to approximately 13.9% based on the Clopper–Pearson exact method. This indicates that the true incidence of ectropion cannot be assumed to be zero, and the results should therefore be interpreted with caution. Second, the absence of a contemporaneous control group within this prospective cohort precludes direct comparison with patients undergoing subciliary incision repair without ADM spacer grafting. Therefore, the observed 0% ectropion rate cannot be definitively attributed to the ADM spacer graft itself, and causal inference remains limited. The findings should be interpreted as preliminary observational outcomes rather than conclusive evidence of efficacy. Accordingly, the results should not be overinterpreted as definitive proof of a preventive effect, but rather as hypothesis-generating observations. Third, the follow-up period was limited to 6 months, and longer-term outcomes beyond this timeframe remain unknown. This relatively short follow-up duration may be insufficient to capture late-onset eyelid malposition or delayed complications related to ADM integration, remodeling, or resorption. Accordingly, the durability of the protective effect observed in this study cannot be fully established. Fourth, the routine use of ADM spacer grafting may introduce additional cost and resource utilization compared with conventional subciliary incision repair without ADM. These economic considerations may limit the widespread adoption of this technique, particularly in healthcare settings with constrained resources. Although the present study suggests a potential benefit in preventing lower eyelid ectropion, the clinical justification for routine ADM use should be interpreted cautiously in the absence of formal cost-effectiveness analysis. Finally, all procedures were performed by a single surgeon, which may introduce operator-related bias and limit external validity. Differences in surgical technique, experience, and perioperative management across institutions may influence outcomes, and therefore the reproducibility of these results in broader clinical settings remains to be validated.

### 4.6. Future Directions

Future research should focus on larger, preferably multicenter studies to validate the preventive effect of ADM spacer grafting. In particular, well-designed comparative studies, including matched cohort analyses or randomized controlled trials comparing subciliary incision repair with and without ADM spacer grafting, are necessary to establish a causal relationship between ADM use and ectropion prevention. Such studies would help determine whether the favorable outcomes observed in this preliminary study can be consistently reproduced in broader and more diverse patient populations. Future investigations should also aim to include sufficiently powered sample sizes to detect clinically meaningful differences in complication rates, including low-incidence events such as ectropion. Long-term follow-up beyond 12 months is warranted to assess the durability of ectropion prevention and scar outcomes. Extended follow-up would be particularly important to evaluate late-onset eyelid malposition and the long-term behavior of ADM, including its integration, remodeling, or potential resorption. Additionally, future studies should incorporate cost-effectiveness analyses to determine whether the potential clinical benefits of ADM spacer grafting justify its additional cost and resource utilization in routine practice. Such analyses would be essential for establishing its role as a standard preventive strategy in subciliary approaches. Finally, histologic and imaging studies examining ADM integration within the eyelid lamellae could further elucidate the mechanisms underlying its protective effects.

## 5. Conclusions

The present study suggests that ADM (WITHderm^®^) spacer grafting during subciliary incision repair for facial fracture surgery may effectively prevent early postoperative lower eyelid ectropion. In this cohort, no cases of ectropion were observed, and both patient- and observer-reported scar outcomes demonstrated significant improvement over time. The interposition of ADM appears to provide structural support to the anterior lamella of the lower eyelid, potentially reducing vertical shortening and cicatricial contracture associated with subciliary approaches.

Given the consistently high rates of ectropion reported in the literature following conventional subciliary incisions, the use of an ADM spacer graft may represent a safe, reproducible, and biologically sound adjunctive technique in selected patients undergoing facial fracture repair. Although these findings are encouraging, larger comparative studies with longer follow-up are required to confirm the durability, generalizability, and cost-effectiveness of this preventive strategy.

## Figures and Tables

**Table 1:** Lower eyelid ectropion grading scale.

Ectropion Degree	Definition
Grade 0 (no ectropion)	No scleral show, no margin eversion
Grade 1 (mild ectropion)	≤2 mm scleral show, no punctal eversion
Grade 2 (moderate ectropion)	>2 mm scleral show, punctal eversion present, mild symptoms (tearing, irritation)
Grade 3 (severe ectropion)	Full-thickness ectropion, conjunctival exposure or keratopathy

**Table 2:** Summary of patient data.

Case No.	Sex/Age	Facial Fracture Type	Ectropion Degree at Postoperative 1 Month
1	F/68	ZMC Fx	Grade 0
2	M/73	Orbital wall Fx	Grade 0
3	M/61	ZMC Fx	Grade 0
4	M/50	Orbital wall Fx	Grade 0
5	M/50	Orbital wall Fx	Grade 0
6	F/34	Orbital wall Fx	Grade 0
7	M/33	ZMC Fx	Grade 0
8	M/62	ZMC Fx	Grade 0
9	F/22	ZMC Fx	Grade 0
10	F/53	ZMC Fx	Grade 0
11	M/62	ZMC Fx	Grade 0
12	F/44	ZMC Fx	Grade 0
13	M/65	ZMC Fx	Grade 0
14	M/65	ZMC Fx	Grade 0
15	M/23	ZMC Fx	Grade 0
16	M/32	ZMC Fx	Grade 0
17	M/57	ZMC Fx	Grade 0
18	M/55	ZMC Fx	Grade 0
19	M/29	ZMC Fx	Grade 0
20	M/69	ZMC Fx	Grade 0

No., number; M, male; F, female; ZMC, zygomaticomaxilliary complex; Fx, fracture.

**Table 3:** Patient and Observer Scar Assessment Scale (POSAS) data of patients.

Case No.	Total PSAS-3M	Total OSAS-3M	OPS-3M	OSR-3M	Total PSAS-6M	Total OSAS-6M	OPS-6M	OSR-6M
1	21	21	4	4	11	12	2	3
2	25	20	4	4	12	13	2	3
3	23	21	5	4	13	11	3	3
4	17	18	3	3	11	12	2	3
5	17	18	3	3	11	12	2	3
6	19	20	4	4	10	10	2	2
7	25	23	5	5	11	11	2	2
8	23	21	5	4	11	10	3	2
9	20	23	4	5	13	10	3	2
10	21	24	5	5	9	10	2	2
11	24	24	5	5	11	14	2	3
12	22	21	5	4	12	13	3	3
13	26	23	5	5	18	14	4	3
14	23	27	5	6	10	14	2	3
15	20	21	4	4	13	10	3	2
16	17	19	3	4	9	10	2	2
17	19	20	4	5	12	12	3	3
18	20	19	4	4	12	12	3	3
19	17	23	4	5	8	8	2	2
20	21	21	4	4	9	10	2	2

No., number; PSAS, patient scar assessment scale; 3M, at 3-month follow-up; OSAS, observer scar assessment scale; OPS, overall patient satisfaction; OSR, objective scar rating; 6M, at 6-month follow-up.

**Table 4:** Comparison of patient scar assessment scale (PSAS) and overall patient satisfaction (OPS) at 3 and 6 months postoperatively.

PSAS	PSAS-3M (Mean ± SD)	PSAS-6M (Mean ± SD)	*p*-Value
Pain	3.0 ± 0.73	1.4 ± 0.50	<0.001
Itching	2.1 ± 0.55	1.1 ± 0.31	<0.001
Color	2.75 ± 0.72	1.75 ± 0.55	<0.001
Stiffness	4.4 ± 0.60	2.4 ± 0.50	<0.001
Thickness	4.5 ± 0.51	2.8 ± 0.62	<0.001
Irregularity	3.55 ± 0.51	1.95 ± 0.51	<0.001
Total score	21.0 ± 2.85	11.3 ± 2.13	<0.001
	OPS-3M	OPS-6M	
OPSS	4.09 ± 1.02	2.37 ± 0.70	<0.001

PSAS, patient scar assessment scale; PSAS-3M, patient scar assessment scale at 3-month follow-up; PSAS-6M, patient scar assessment scale at 6-month follow-up; OPS, overall patient satisfaction; OPS-3M, overall patient satisfaction at 3-month follow-up; OPS-6M, overall patient satisfaction at 6-month follow-up; SD, standard deviation.

**Table 5:** Comparison of observer scar assessment scale (OSAS) and objective scar rating (OSR) at 3 and 6 months postoperatively.

OSAS	OSAS-3M (Mean ± SD)	OSAS-6M (Mean ± SD)	*p*-Value
Vascularity	2.0 ± 0.00	1.1 ± 0.31	<0.001
Pigmentation	2.0 ± 0.00	1.2 ± 0.41	<0.001
Thickness	4.45 ± 0.51	2.30 ± 0.47	<0.001
Pliability	3.65 ± 0.59	2.05 ± 0.39	<0.001
Relief	3.70 ± 0.57	1.85 ± 0.37	<0.001
Surface area	4.00 ± 0.65	2.10 ± 0.45	<0.001
Total score	21.35 ± 2.25	11.4 ± 1.67	<0.001
	OSR-3M (mean ± SD)	OSR-6M (mean ± SD)	
OSR	4.19 ± 1.05	2.46 ± 0.65	<0.001

OSAS, observer scar assessment scale; OSAS-3M, observer scar assessment scale at 3-month follow-up; OSAS-6M, observer scar assessment scale at 6-month follow-up; OSR, objective scar rating; OSR-3M, objective scar rating at 3-month follow-up; OSR-6M, objective scar rating at 6-month follow-up; SD, standard deviation.

## Data Availability

The original contributions presented in this study are included in the article. Further inquiries can be directed to the corresponding author.

## Abbreviations

The following abbreviations are used in this manuscript:

ADM	Acellular dermal matrix
ZMC	Zygomaticomaxillary complex
POSAS	Patient and Observer Scar Assessment Scale
PSAS	Patient Scar Assessment Scale
OSAS	Observer Scar Assessment Scale
OPS	Overall patient satisfaction
OSR	Objective scar rating
SD	Standard deviation
